# Bone Sialoprotein Shows Enhanced Expression in Early, High-Proliferation Stages of Three-Dimensional Spheroid Cell Cultures of Breast Cancer Cell Line MDA-MB-231

**DOI:** 10.3389/fonc.2019.00036

**Published:** 2019-02-05

**Authors:** Valeh Rustamov, Florian Keller, Julia Klicks, Mathias Hafner, Rüdiger Rudolf

**Affiliations:** ^1^Institute of Molecular and Cell Biology, Mannheim University of Applied Sciences, Mannheim, Germany; ^2^Institute of Medical Technology of Heidelberg University and Mannheim University of Applied Sciences, Mannheim, Germany

**Keywords:** BSP, bone sialoprotein, breast cancer, MDA-MB-231, spheroid, 3D cell culture, proliferation, apoptosis

## Abstract

Normally, bone sialoprotein (BSP) is an important contributor to bone micro-calcification. However, it is also highly expressed in bone-metastatic malignancies, including prostate, lung, and breast cancer. In these disorders, BSP correlates with poor prognosis. Its expression in triple-negative breast cancer cells is enhanced by the transcription factor RUNX2, and both, BSP and RUNX2 are under control of IGF-1 and TGFβ1. Knockdown of BSP or its inactivation by specific antibodies were found to reduce the metastatic potential of MDA-MB-231 triple-negative breast cancer cells in xenografts. While the role of BSP in bone metastasis was studied using such *in vivo* models, valid *in vitro* test systems to investigate BSP biology have been lacking since this protein is expressed at very low levels in classical 2D cell cultures and the frequently used breast cancer cell line MDA-MB-231 is difficult to grow in 3D. Here, we have developed a long-term 3D spheroid culture model using MDA-MB-231 cells in a sandwich approach using cell embedding between a non-adherent surface and basement membrane extracts. This allowed consistent growth of spheroids for more than 21 days. Also, co-culturing of MDA-MB-231 with CCD-1137Sk fibroblasts yielded stably growing spheroids, suggesting the importance of extracellular matrix (ECM) in this process. In addition, we have set up a novel and simple open source analysis tool to characterize protein expression in 2D cultures and spheroids by immunofluorescence. Using this approach in combination with Western blot analysis, the expression profile of BSP was analyzed. BSP was enriched at the rims of spheroids, both in mono- and co-cultures and its abundance in general correlated with that of TGFβ1 under different conditions, including spheroid maturation, cytostatic treatment, and fibroblast co-culture. Conversely, correlation of IGF-1 and BSP was limited to mono-culture time course profiles. In conclusion, we present novel tools to study the regulation of gene expression in combination with cell proliferation and apoptosis in a long-term 3D model of breast cancer and find dynamic abundance profiles of the metastasis-relevant protein BSP and its regulators.

## Introduction

Breast cancer is the most frequent neoplastic lesion in women. When associated with distant metastasis, the overall prognosis for breast cancer is poor with a 5-years survival in stage IV of about 27% (1). Breast cancer can be subdivided into four molecular subtypes: positive for either luminal A, luminal B, or Her-2, and triple negative (2, 3). Metastasis is frequent in breast cancer and typically affects liver, bone, lung, or a combination of these (3–5), with bone being the most frequently targeted organ of breast cancer metastasis (6). This might be due to bone's rich depots of nutrients, growth factors (TGFβ, IGF, VEGF, M-CSF, FGF, MCP, BMP2) and fine blood supplies (7). Furthermore, bone contains a special type of capillaries called sinusoids, which are characterized by slow blood circulation and porous endothelial walls, that facilitate the extravasation of metastatic cells into the bone marrow (8). Mortality is positively correlated with bone metastasis (7, 9–13) and develops in 65–75% of patients with advanced breast cancer (14). Metastatic injury of bone leads to failure of bone homeostasis (15). Indeed, while bone remodeling processes (16) are normally characterized by a balance between osteoblast and osteoclast activities, breast cancer metastases often stimulate the osteolytic process (17). Metastatic cancer cells produce special enzymes, such as matrix metalloproteases (MMPs), cytokines (IL-6, IL-8, IL-11), parathyroid hormone-related protein (PTHrP), chemokine receptor (CXCR4), osteopontin (OPN), and bone sialoprotein (BSP) which help to invade bone marrow (18–24). In most cases, bone metastases are associated with bone pain, hypercalcemia, pathologic fractures, spinal cord instability, and total bone marrow infiltration. Furthermore, progression of tumor invasion into bone marrow and long-term issues of chemo- and radiation therapy complicate blood diseases such as anemia, neutropenia, leukopenia, and pancytopenia (25, 26). Therefore, strategies which could reduce the incidence and morbidity of bone metastases are of great clinical importance.

BSP is a non-collagenous phosphorylated glycoprotein which was originally isolated from calf bone (27). It is a member of the SIBLING (Small Integrin-Binding Ligand, N-linked Glycoprotein) protein family which also contains osteopontin (OPN), dentin sialophosphoprotein (DSPP), dentin matrix protein 1 (DMP1), and matrix extracellular phosphoglycoprotein (MEPE). Generally, SIBLING proteins function in adhesion, migration, and spreading of cells through interaction with multiple binding partners such as MMPs, CD44, and integrins (28, 29). Normally, they exert functions not only in mineralized tissues such as bone and dentin (30), but in soft organs, too (31, 32). However, in cancer they are differentially regulated in tumor invasion, cell survival and proliferation. This suggests an essential role of SIBLING proteins in tumorigenesis and cancer progression (33). Accordingly, BSP is not only expressed in healthy bone, cartilage, teeth, and trophoblasts of the placenta but also in primary and secondary tumors (34, 35). It is used as an early marker for osteoblast differentiation (36) and accelerates differentiation of mesenchymal cells from bone into osteoblasts (37). On SDS-PAGE, BSP, with a molecular mass of the core protein of 33.6 kDa, runs at 70–80 kDa due to glycosylation. These smaller and larger forms were termed hypo-glycosylated BSP (hypo-BSP) and high-glycosylated or mature BSP (mature-BSP), respectively (38). Moreover, literature has also shown a band at 45–52 kDa (38, 39). BSP contains an RGD integrin recognition sequence which may facilitate adhesion of tumor cells to the bone surface, especially through α_v_β_3_ and α_v_β_5_ integrin receptors (40, 41). Beyond that, the RGD-integrin complex was found to interact with MMP-2 and human complement factor H, which mediates a block of tumor cell lysis during metastasis (42, 43). Therefore, patients with preoperatively elevated serum BSP levels are at high risk of subsequent bone metastases in the 1st years after primary surgery (44). Thus, there seems to be a connection between ectopically formed BSP and the development or progression of osseous metastasis in breast cancer. Indeed, in primary breast cancer, BSP expression correlated with a bad prognosis and the development of bone metastasis (45). Downregulation of BSP through antisense oligonucleotides reduced the formation of colonies of MDA-MB-231 breast cancer cells and of osteolytic metastases in nude rats (46). Furthermore, use of an anti-BSP antibody led to decreased proliferation, colony formation, and migration of breast cancer cells *in vitro* and reduced osteolysis in a nude rat cancer model (47). These findings suggest that BSP plays an important role in breast cancer bone metastasis and might serve as a useful marker protein. Expression of BSP is mediated by the transcription factor RUNX2 (48). RUNX2 expression, in turn, is regulated by TGFβ1 (49, 50) and its DNA-binding activity appears to be induced by ERK- and/or AKT-dependent phosphorylation as a consequence of IGF-1 binding (51, 52). Fittingly, BSP expression was also found to be downstream of TGFβ1 (53, 54) and IGF-1 (55).

Until today, experiments related to BSP were either performed in conventional two-dimensional (2D) cell cultures or using *in vivo*-rodent models. Although culturing of cells in 2D and their use for studying drug effects are easy to achieve, 2D models show significant limitations in reproducing the complexity and pathophysiology of *in vivo* tumor tissue (56). Therefore, three-dimensional (3D) cell culture systems are of increasing interest in cancer research since tissue architecture and the extracellular matrix (ECM) significantly influence tumor cell responses to micro-environmental signals (57). The 3D systems display several characteristics of tumor cells *in vivo*. These include gradients of oxygen and nutrients, with according cellular subpopulations showing proliferative, quiescent, or apoptotic/necrotic behavior. Consequently, models that better mimic tumor heterogeneity and intercellular contact were found to exhibit more representative responses to drug therapies (58, 59). Here, we set up a sandwich approach and a fibroblast co-culture model for long-term 3D cell culture which allows consistent growth of spheroids made of triple negative MDA-MB-231 breast cancer cells for more than 21 days. Expression profiles of BSP were analyzed in 2D and 3D cell culture systems and a new non-commercial anti-hypo-BSP monoclonal antibody was explored. This revealed a correlation of BSP expression with TGFβ1.

## Materials and Methods

### Cell Line and Cell Culture

The human MDA-MBA-231 breast cancer cell line, the non-cancerous human breast epithelial cell line MCF10A, the prostate cancer cell line PC-3, and the foreskin fibroblast cell line CCD-1137Sk were purchased from American Type Culture Collection (ATCC, Manassas, VA, USA). MDA-MB-231 and PC-3 cells were maintained as monolayers in RPMI 1640 medium with L-glutamine (Capricorn Scientific GmbH, Germany) supplemented with 10% fetal bovine serum (FBS, Gemini Bioproduct Inc., Woodland, CA, USA) and 1% penicillin/streptomycin (Capricorn Scientific GmbH, Germany) at 37°C in an incubator with 5% CO_2_. MCF10A cells were cultured in a 1:1 mixture of Dulbecco's modified Eagle's medium and Ham's F12 medium (DMEM/F12) (Capricorn Scientific GmbH, Germany) supplemented with 5% horse serum (Sigma Aldrich, Germany), hydrocortisone (0.5 μg/ml) (Sigma Aldrich, Germany), insulin (10 μg/ml), epidermal growth factor (20 ng/ml) (Sigma Aldrich, Germany), and 1% penicillin-streptomycin. The medium was changed every 2–4 days. After the monolayer of cells became 80% confluent, sub-cultivation was carried out with using 0.05% Trypsin-EDTA in DPBS (1x) (Capricorn Scientific GmbH, Germany). CCD-1137Sk cells were maintained as monolayers in Iscove's Modified Dulbecco's Medium (ATCC, Manassas, VA, USA) supplemented with 10% FBS and 1% penicillin/streptomycin (Capricorn Scientific GmbH, Germany) at 37°C in an incubator with 5% CO_2_.

### Tumor Spheroid Formation and Cytostatic Treatment

The cell numbers of monolayer cell cultures were determined using a Vi-CELL XR cell counter and cell viability analyzer (Beckman Coulter, Fullerton, CA). MDA-MB-231 and PC-3 three-dimensional mono-cultures were generated using four different methods described below and summarized in Table 1. In order to examine the effects of different 3D techniques on long term culture, cells were seeded at day *in vitro* (DiV) 0 with 10,000 cells per well. For co-cultures of MDA-MB-231 with CCD-1137Sk cells, 10,000 cells of each type were mixed and then co-seeded on ultralow attachment U-bottom plates (Corning, Corning, NY, USA) in MDA-MB-231 medium. Then, plates were centrifuged for 5 min at 500 × g. For cytostatic treatment, 6 days old spheroids were cultivated for 48 h in either 1 μM Paclitaxel (Sigma Aldrich, Germany) in 0.5% of DMSO or just in 0.5% of DMSO as control. Finally, samples were harvested, fixed, and prepared to staining.

**Table 1:** Overview of experimental 3D culture design.

**Method**	**Subgroup**	**Initial seeding density**	**Medium**	**Coating type**
Hanging drop technique (HD)	–	10,000 cells/well	RPMI 1640	–
Inlay method (IM)	IM1	10,000 cells/well	Methylcellulose 0,3%	–
	IM2		RPMI 1640	Cell repellent surface
	IM3		Methylcellulose 0,3%	Cell repellent surface
On top method (OM)	OM1	10,000 cells/well	RPMI 1640	Methylcellulose 1,5%
	OM2			Sea plaque agarose 1,5%
Sandwich method (SM)	SM1	10,000 cells/well	RPMI 1640 + Matrigel 10%	Methylcellulose 1,5%
	SM2			Sea plaque agarose 1,5%
	SM3		RPMI 1640 + BME 10%	Sea plaque agarose 1,5%
	SM4	5,000 cells/well		Sea plaque agarose 1,5%

#### Hanging Drop Technique (HD)

Twenty microliter of cell suspension per well were applied into a 72-well Terasaki plate from Greiner Bio-One, Germany. The hanging drop plate was then carefully rotated upside down and placed into a 100 mm × 20 mm plate. Into the same plate also a 60 mm tissue culture dish without lid was placed and supplied with 5 ml of double-distilled water (ddH2O) on the bottom of the dish to keep the humidity in the plate constant. At the end, the lid of the 100 mm × 20 mm plate was closed and incubated at 37°C in a humidified atmosphere at 5% CO_2_. Daily monitoring of the 3D cell cultures was performed after four days under an inverted phase-contrast microscope (Axiovert 25, Zeiss). Medium was changed every other day by adding 2.5 μl fresh medium per well.

#### Inlay Method (IM)

This method was essentially performed as described before in detail (60). Briefly, 7.2 g of methylcellulose (MC) powder (Sigma-Aldrich, Germany) were autoclaved together with a magnetic stirrer. Three hundred milliliter of 60°C pre-warmed RPMI 1640 medium were added to the MC powder, the resulting MC solution was stirred for 20 min. Thereafter, 20% FCS were added, and the solution was mixed again overnight at 4°C under sterile conditions. The solution was aliquoted in 15 ml tubes, centrifuged at 5,000 × g for 2 h at 23°C, and the supernatant was stored at −20°C. The corresponding cell suspension was mixed well at the rate 4:1 with pre-warmed MC solution at room temperature and 150 μl cell suspension was pipetted into 96-well plates (Greiner Bio-one, Frickenhausen, Germany). The final concentration of MC was 0.36% per well, respectively. Spheroid formation was induced by centrifugation of the plates at 800 × g for 15 min and incubated in a humidified atmosphere at 37°C and 5% CO_2_. For subgroups IM2 and IM3 (see Table 1) cell-repellent surface plates (CELLSTAR®) were used. In all groups, the 3D cell cultures were observed for 24 DiV of culturing and medium was changed every other day by replacing 50% of the medium.

#### On-Top Method (OM)

To avoid cell attachment to the plate bottom and to stimulate 3D cell culture generation, 96-well f-bottom plates (Greiner Bio-one, Frickenhausen, Germany) were coated with pre-warmed SeaPlaque® GTG (Cambrex Bio Science Rockland, Rockland, ME) agarose or with MC solution as described below. For the OM1 subgroup the MC 1,5% solution was prepared as described for the IM method, but FCS was not included. For the OM2 subgroup, sea plaque agarose (SPA) was diluted in RPMI 1640 medium with l-glutamine without FCS to a final concentration of 1.5% and then autoclaved together with a magnetic stirrer. Thereafter, for the OM1 subgroup 96-well f-bottom plates (Greiner Bio-one, Frickenhausen, Germany) were coated with 50 μl per well of pre-warmed 1.5% MC solution. For the OM2 subgroup 96-well f-bottom plates (Greiner Bio-one, Frickenhausen, Germany) were coated with 50 μl per well of pre-warmed 1.5% SPA solution, respectively. After the first layer had been allowed to solidify, a single-cell suspension containing 10^4^ cells per 150 μl was plated in complete growth medium into each well. The plates were centrifuged at 800 rpm for 15 min to allow cell-to-cell contact and incubated in a humidified atmosphere at 37°C and 5% CO_2_. The 3D cell cultures were observed for 24 DiV of culturing and medium was changed every other day by replacing 50% of the medium.

#### Sandwich Method (SM)

Here, we describe an improved version of a technique that was previously published by others (61–64). The first layer for the sandwich technique was prepared as described for the OM method. The plates were then allowed to cool down under laminar flow for 60 min at room temperature. Thereafter, 50 μl of cell suspension containing 10^4^ cells were added per well and the plates were then centrifuged at 800 rpm for 10 min to initiate spheroid formation. As basement membrane-like extracellular matrix extracts, we used Matrigel (Cat. No: 354234, BD Biosciences) or BME (Cat. No: 3445-010-01, Cultrex® 3-D Culture Matrix™ Reduced Growth Factor Basement Membrane Extract, PathClear®, Amsbio). Matrix stock solutions were thawed overnight on ice at 4°C and then mixed on ice with cell culture medium to reach a final concentration of 10%. Fifty microliter of 10% Matrigel or BME solution were then gently added to each well. Subsequently, spheroids were cultured statically under standard culture conditions (5% CO_2_, 37°C). The spheroids were observed for 24 days of culturing and medium was changed every other day by replacing 50% with fresh medium.

### Analysis of Tumor Spheroids

To determine the long-term growth kinetics of 3D cell cultures under each condition, the spheroid/aggregate sizes were examined at DiV 4–24 using an inverted phase-contrast microscope (Axiovert 25, Zeiss). The digitalized images were then processed and analyzed by measuring the area of the 3D cell cultures using ImageJ (V 1.48) software. In contrast, many previous studies reported the volumes of spheroids (62, 65–68). These volume values were mostly derived from measured areas, diameters, or perimeters of the spheroids in the 2D projection. Such a procedure might be useful for perfectly round-shaped 3D cultures. However, here we obtained many non-circular 3D cell aggregates and this made it impossible to get the volume values for 3D cultures. Therefore, we measured the areas of 3D cultures to compare morphological characteristics and growth kinetics.

### Immunoblotting

For immunoblotting, about 180–190 spheroids were accumulated in 15 ml conical tubes and allowed to sediment. Supernatant was discarded by gentle aspiration and the spheroids were washed twice with PBS (Capricorn Scientific GmbH, Germany). After washing, all spheroids were transferred to an ice cold 0.1 ml micro tissue grinder glass (Wheaton, USA) and 100 μl of NP-40 lysis buffer (PMFS 1:100; inhibitor cocktail 1:100) were added. The spheroids were grinded manually and incubated for 15 min on ice. Afterwards, lysates were centrifuged at 10,000 rpm for 15 min at 4°C and the supernatants stored at −25°C. Cell lysates from 2D cultures were also prepared using 100 μl NP-40 lysis buffer. Protein concentrations were measured using a BCA Protein Assay kit (Pierce). After determining protein concentration, samples were mixed with 2 × Laemmli buffer (69) without 2-Mercaptoethanol and boiled for 5 min. Thirty microgram of protein and standard marker (NIPPON Genetics Europe, Germany) were resolved on 8% SDS-PAGE, transferred to nitrocellulose transfer membrane (Protran®, Schleicher and Schuell Bioscience GmbH) and blocked with 5% milk in TBS-Tween 20 (0.1%, Sigma). The membranes were probed with primary antibodies for 16 h at 4°C and then incubated with HRP-conjugated secondary antibodies for 2 h at room temperature. Non-commercial human recombinant carbohydrate deficient bone sialoprotein [CD-BSP (aa 108–122)] and commercially available rat CD-BSP (Cat. No: 4217.VP) primary monoclonal antibodies (mAb) were received as gifts from Immundiagnostik AG, Germany. Additional information about antibodies and dilutions used in the study are shown in Supplementary Table 1. Blots were visualized by chemiluminescence (Westernbright chemiluminescent substrate sirius; Biozym Scientific GmbH) and imaged using the Syngene G-Box (Syngene, Frederick, MD, USA). Band intensities were quantified using the analysis software ImageJ as relative intensities of bands of interest divided by the intensities of the corresponding GAPDH bands.

### Immunofluorescence

For immunofluorescence, spheroids were accumulated in 15 ml conical tubes and allowed to sediment. Supernatant was discarded by gentle aspiration and the spheroids were washed twice with sterile PBS. After washing, 4–5 spheroids were transferred to a 1.5 ml tube. After sedimentation, the supernatant was gently removed and 4% paraformaldehyde (PFA) was added and placed on an orbital shaker at 1,000 rpm for 2 h at room temperature. Then, PFA was removed and spheroids were washed twice with PBS. Afterwards, PBS was removed, spheroids were soaked in 10% sucrose and placed on an orbital shaker at 1,000 rpm for 15 min at room temperature. After 15 min, the sucrose was gently aspirated and then new 10% sucrose solution was added. Subsequently, a sucrose gradient was applied by incubating 15 min each in increasing sucrose densities (mix 2:1; 1:1; 1:2 with 30% sucrose). To prepare cryosections, 4–5 spheroids were transferred into Tissue-Tek (Cryomold®), embedded with frozen section compound (Leica) and frozen at −80°C. The blocks were cut on a cryostat (Leica CM 1950) at 10 μm thickness. The slides were kept frozen at −80°C until being stained. Monolayer cell cultures were fixed with 4% PFA for 15 min and then washed twice with PBS. Fixed sections were permeabilized with 0,1% Triton X-100 for 5 min and then washed twice with PBS. To avoid unspecific staining, the sections were incubated with blocking solution (10% horse serum, 0.2% fish skin gelatin in 1 × PBS) and incubated for 20 min at room temperature. Then, sections were incubated with the primary antibodies overnight at 4°C in a humidified chamber. Sections were then washed twice with blocking solution for 20 min each. Then, they were incubated with secondary antibodies for 1.5 h at room temperature and subsequently washed twice with blocking solution and PBS. After drying, the samples were covered with 10% Mowiol® (Sigma Aldrich) and allowed to dry overnight in dark storage boxes at room temperature. Slides were imaged on a Leica SP8 confocal laser-scanning microscope with HP PL APO 20x/0.75 IMM CORR oil immersion objective. The antibodies and dyes used in this study are summarized in Supplementary Table 1.

### Analysis of Confocal Images

All images were analyzed using the image processing software ImageJ (https://imagej.nih.gov/ij/). For automated image-based analysis and to generate high accuracy of measurements, we developed a multistep segmentation routine on the basis of ImageJ (Data Sheet 1). This comprised of the following steps (Supplementary Figure 1). First, images were duplicated and on these, noise-pixels were removed with a median filter (radius 1.0). Images were adjusted for brightness/contrast to distinguish relevant structures against the background. Resulting images were binarized and dilated. Next, these binarized images were processes with auto threshold, watershed, and all objects larger than 50 pixels were detected using the analyze-particles function and imported into the ROI manager. Afterwards, all images were checked by visual inspection. Furthermore, a manual check was done by detection of unspecific ROIs, which were located outside of the spheroid sections. Subsequently, all ROIs were overlaid on the original raw file and measurements were performed on these without any quality loss. Finally, all data were calculated with Microsoft ® Excel for Mac OS (Version 16.0). For determination of enrichment of BSP in outer vs. inner cells of co-cultures, each spheroid was separated into an inner area (= inside) and an outer area (= outside). The BSP channel was segmented and the sum of the total spheroid signal and the sum of the outside signal was measured. Each sum was then divided by the number of total ROIs and outside ROIs, respectively, to get the mean of total spheroid BSP signal and outside BSP signal, respectively. The sum of the inside signal was measured by the sum of total signal minus the sum of outside signal. The mean of the inside BSP signal was then measured by dividing the sum of inside signal by the number of inside ROIs.

### Statistical Analysis

Statistical analysis employed Graph Pad Prism V7.0 (Graph Pad Software Inc., USA). Two-way ANOVA with Tukey's multiple comparisons *post hoc* test (confidence level 95%) was used for comparison among 3D culture systems and growth kinetic of MDA-MB-231 spheroids/aggregates by considering the different 3D cell culture methods and the time as two factors. Statistical significance of data from western blot and immunofluorescence experiments was evaluated using one-way Analysis of Variance (ANOVA) with Tukey's *post hoc* test or using unpaired two-tailed *t*-tests. Normal distribution and homo/heteroscedasticity of data were probed using Kolmogorov-Smirnov test and *F*-test, respectively. Bar graphs are presented as mean ± standard deviation (SD). *P*-values were indicated as ^*^ (*P* < 0.05), ^**^ (*P* ≤ 0.01), ^***^ (*P* ≤ 0.001) or ^****^ (*P* ≤ 0.0001) and *P* > 0.05 was considered not significant (“ns”).

## Results

### Influence of 3D Cell Culture Protocols on Aggregate/Sphere Formation and on Growth of MDA-MB-231 Cells

**Figure 1:**
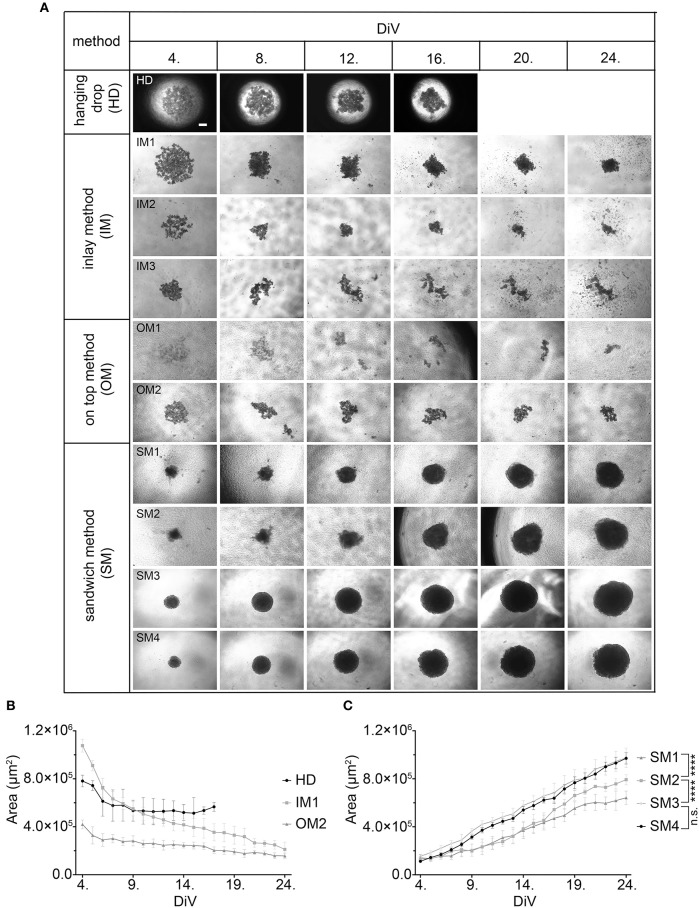
Sandwich method using SPA and BME yields most consistent growth of MDA-MB-231 spheroids. **(A)** Representative phase-contrast microscopy images of MDA-MB-231 3D cultures. These were generated using four different methods: hanging drop (HD), inlay method (IM1; IM2; IM3), on top method (OM1; OM2), and sandwich method (SM1; SM2; SM3; SM4). From HD to SM3, equal initial seeding densities were used (10,000 cells/well), for SM4 5,000 cells/well were used. Spheroids were cultured until day 24 *in vitro* (DiV). Representative images from DiV 4, 8, 12, 16, 20, and 24 are shown. Scale bars, 200 μm. **(B,C)** Quantitative analysis of 3D culture growth or shrinkage. Graphs depict areas of 3D cultures as a function of DiV (mean values ± SD, *n* = 4 independent experiments with 5 replicates). Statistical analysis was performed using a two-way ANOVA with *post-hoc* Tukey's multiple comparison test to compare statistically significant differences across methods for each day. Statistical significance values were observed for 24 DiV: (SM1 vs. SM2, ^****^P < 0.0001; SM2 vs., SM3 ^****^P < 0.0001; SM3 vs. SM4, n.s.).

First, we compared and refined different previously described 3D culture protocols for MDA-MBA-231 cells to develop a consistent, reproducible long-term spheroid culture. Therefore, cells were grown under four different conditions, i.e., hanging drop (HD), inlay (IM), on top (OM), and sandwich methods (SM) (for details of culture conditions, refer to Materials and Methods section and Table 1). Figure 1A shows representative microscopic images to illustrate morphological changes of 3D cell aggregates and spheroids during an observation time period of 24 DiV. A qualitative and quantitative analysis of these cultures revealed the following results. First, cells in HD, IM, and OM cultures did not form spheroids (Figure 1A) but remained loose cell aggregates that decreased in size during the observation period of 24 DiV (Figure 1B). Cell spreading was pronounced in IM cultures (Figure 1A) and low in HD and OM aggregates (Figure 1A). In contrast to HD, IM, and OM subgroups, we observed formation of regularly round spheroids (Figure 1A) and their permanent growth (Figure 1C) in all SM subgroups. Spheroids sandwiched between 1.5% SPA and 10% Matrigel (SM2) grew 1.3-fold faster than spheroids located between 1.5% MC and 10% Matrigel (SM1). Moreover, cells coated with 10% BME (SM3 and SM4) showed higher linear expansion of spheroids than those with 10% Matrigel (SM1 and SM2). To determine the best linear growth and the optimal size of spheroids embedded with 10% BME, cells were seeded at different seeding densities. Initial seeding densities of 5,000 cells per well (SM4) and 10,000 cells per well (SM3) resulted in similar growth curves of spheroids (Figure 1C). The borders of SM3-4 spheroids were clearly visible and of round shape in comparison with spheroids of conditions SM1 and SM2. Thus, SM3 yielded the most consistent and clearly defined spheroids. To verify a more general applicability of the SM3 culture protocol, this was applied to prostate cancer PC-3 cells, another cell line that is difficult to culture in a spheroid format. As depicted in Supplementary Figure 2, this led to regularly round and consistently growing spheroids similar to those of MDA-MB-231 SM3 cultures.

### Spheroid Formation Upon Co-culturing of MDA-MB-231 and CCD-1137Sk Fibroblasts

**Figure 2:**
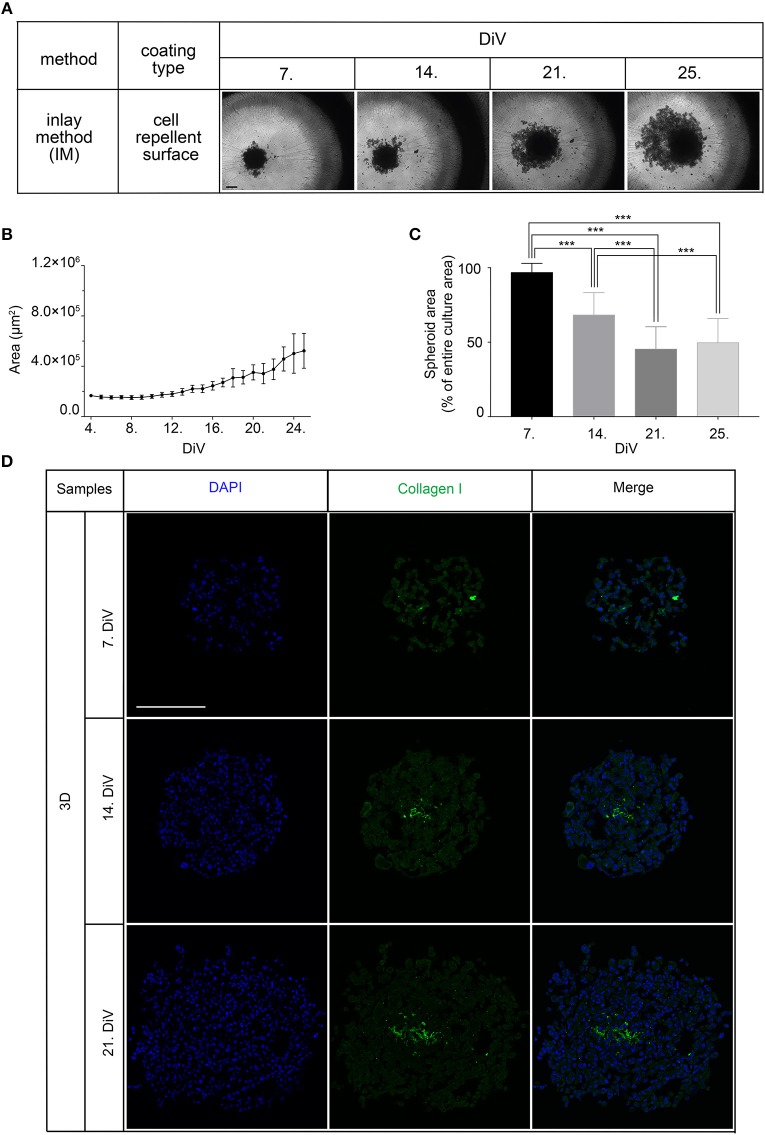
Co-culture of MDA-MB-231 and CCD-1137Sk fibroblast cells yields slowly growing spheroids and extensive outward cell movement. **(A)** Representative phase-contrast microscopy images of MDA-MB-231 plus CCD-1137Sk 3D co-cultures from 7, 14, 21, and 25. DiV. These were generated by co-seeding of 10,000 cells for each type in ultra-low attachment plates. Scale bar, 200 μm. **(B)** Quantitative analysis of 3D culture growth. Graphs depict average values of dark compact spheroid areas and excluding the outward spreading cell region, as a function of DiV (mean values ± SD, *n* = 96 spheroids). **(C)** Quantitative assessment of outward moving cells. Columns indicate the fraction of the total culture area that was occupied by the dark compact spheroid structures in percent and as a function of DiV (mean values ± SD, *n* = 96 spheroids; ^***^P < 0.001). **(D)** Slices of co-culture spheroids at 7., 14., and 21. DiV were stained with DAPI for nuclei and anti-Collagen I antibody for ECM. Panels show single confocal sections of representative samples. Scale bar, 200 μm.

Given that BME as well as Matrigel are rich in ECM components, we wanted to test the effects of ECM supply by fibroblast co-culture. Therefore, 10,000 MDA-MB-231 cells were co-seeded with an equal amount of CCD-1137Sk foreskin fibroblasts in the absence of BME and Matrigel on ultra-low attachment plates. Appearance and growth of the resulting cultures were studied. As shown in Figure 2A, these co-cultures formed more or less round 3D cultures of spheroid shape, and they were growing with increasing culture time (Figure 2B). However, in contrast to SM3 monocultures of MDA-MB-231 cells, which formed smoothly outlined and almost perfectly round spheroids and were already consistently growing after 4. DiV (Figures 1A,C), the co-cultures displayed a more rugged surface with individual cells outside the spheroid (Figure 2A) already at 7. DiV. The amounts of cells not adhering to the spheroid increased with culture time and by 21. DiV only about 50% of the area occupied by the entire co-culture was really found within the spheroid (Figure 2C). In other terms, these co-cultures increasingly disintegrated with culture time. Notably, collagen I as a major ECM component was found at all spheroid stages throughout the co-cultures, but often enriched in a central region (Figure 2D). In summary, these data suggest that ECM is a critical component for formation and growth of MDA-MB-231 spheroids but it appears to be insufficient for maintaining the spheroids compact.

### Differential Expression of Mature and Hypo-BSP in 3D vs. Monolayer Cell Cultures

**Figure 3:**
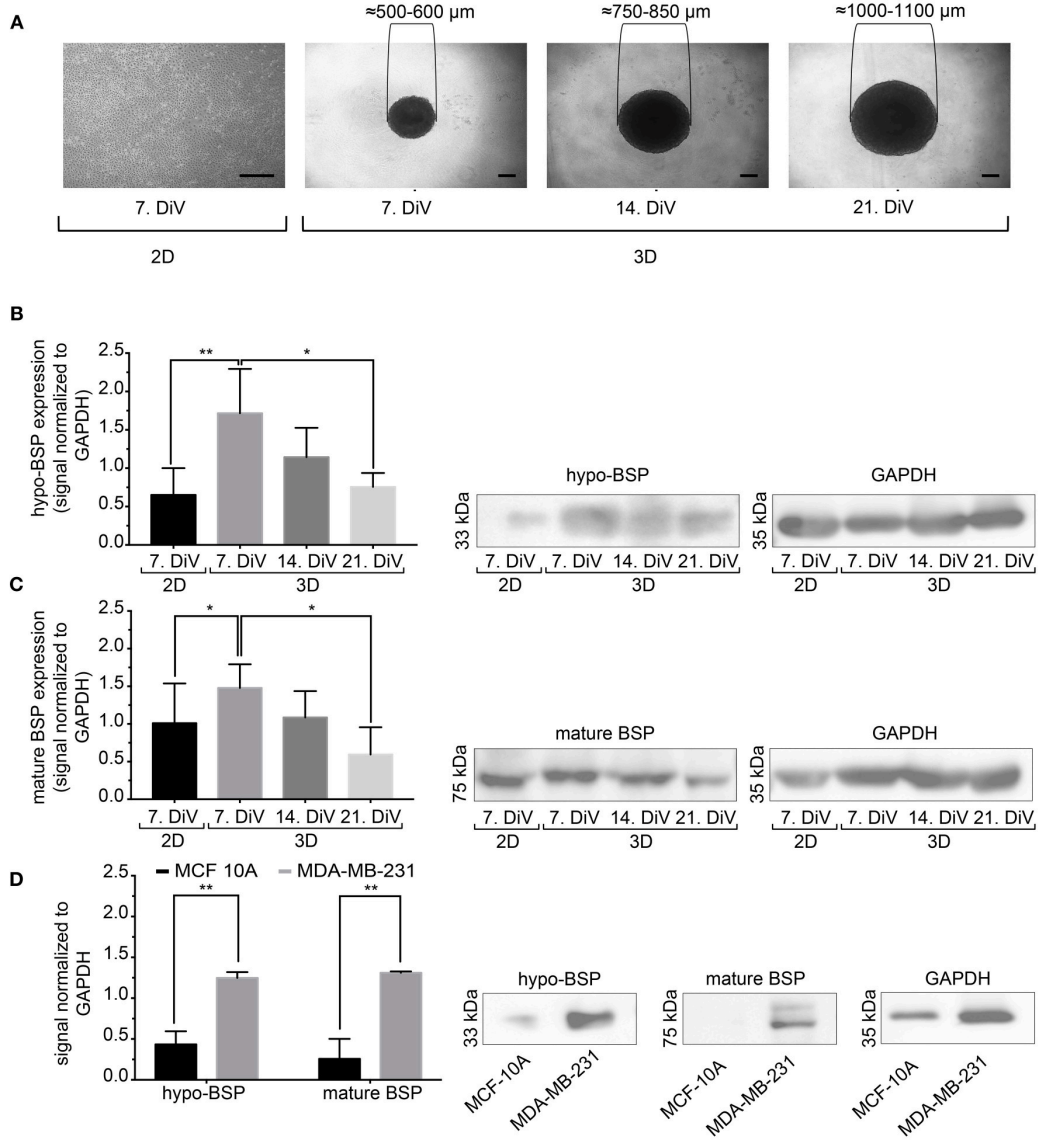
Mature and hypo-BSP protein levels are increased in 3D vs. 2D cultures at 7. DiV. **(A)** Wide field images of representative 7. DiV monolayer cells and 7.,14., and 21. DiV spheroids as used for biochemical analyses. Scale bars, 200 μm. **(B,C)** Equal amounts of protein of lysates from monolayer cells (2D) and spheroids (3D) were loaded on SDS-PAGE and Western blots were performed. Left panels depict quantitative analyses of Western blot bands as shown for representative cases in right panels. **(B)** Results upon incubation with human anti-recombinant human CD-BSP (aa 108–122) showing hypo-BSP expression. Statistical analysis was performed with one-way ANOVA followed by Tukey's multiple comparisons test (*n* = 6, ^**^P < 0.01, ^*^P < 0.05, mean ± SD). **(C)** Results upon incubation with rat anti-recombinant human CD-BSP (aa 108–122) TGC-9 indicating expression of mature BSP. Statistical analysis was performed with one-way ANOVA followed by Tukey's multiple comparisons test (*n* = 6, ^*^P < 0.05, mean ± SD.). **(D)** Lysates of non-cancerogenous breast cell line MCF 10A were compared to those of MDA-MB-231. Graph shows quantitative analysis of Western blot band intensities as exemplified on the right side. Significant differences between groups were tested using an unpaired, two-tailed *t*-test (*n* = 3, ^**^P < 0.01, mean ± SD).

Next, we studied the kinetic profile of BSP expression at different times in SM3 MDA-MB-231 monocultures. Therefore, 2D cells were harvested at 7. DiV and spheroid cultures at 7., 14., and 21. DiV for Western blot analysis. Figure 3A shows representative wide field images of these samples. For immunoblotting, 180–190 spheroids were lysed for each trial on 7., 14., and 21. DiV. For each time point, spheroids showed rather homogeneous sizes (Figure 3A). In the Western blot experiments, we validated a novel human anti-recombinant human CD-BSP (aa 108–122) mAb against BSP and compared its performance with a commercially available rat anti-recombinant human CD-BSP (aa 108–122) TGC-9 mAb. The human CD-BSP mAb detected a band at the expected size for hypo-BSP at 33 kDa, as well as a lower one at 25 kDa (Supplementary Figure 3). Conversely, the rat CD-BSP mAb primarily detected a band at 75 kDa, which likely reflected mature BSP, and additionally some weak bands at sizes of 45–50 kDa and 35 kDa, which likely reflected partially glycosylated forms of BSP (Supplementary Figure 3). To examine the specificity of both antibodies, we used the non-cancerous human breast epithelial cell line MCF-10A as a negative control. This showed a highly significant difference in the expression of both BSP forms. While BSP-specific bands were hardly detectable in MCF-10A lysates they were strong in MDA-MB-231 samples (Figure 3D). Comparison of different MBD-MB-231 cultures revealed that hypo-BSP and mature-BSP expression was significantly higher in 7. DiV 3D cultures as compared to 2D monolayer cultures (Figures 3B,C). With increasing time in 3D cultures, expression of hypo-BSP and mature-BSP decreased and it was significantly lower on 21. DiV as compared to 7. DiV (Figures 3B,C).

### Increased Expression of BSP in Young MDA-MB-231 Spheroids as Revealed by Immunofluorescence

**Figure 4:**
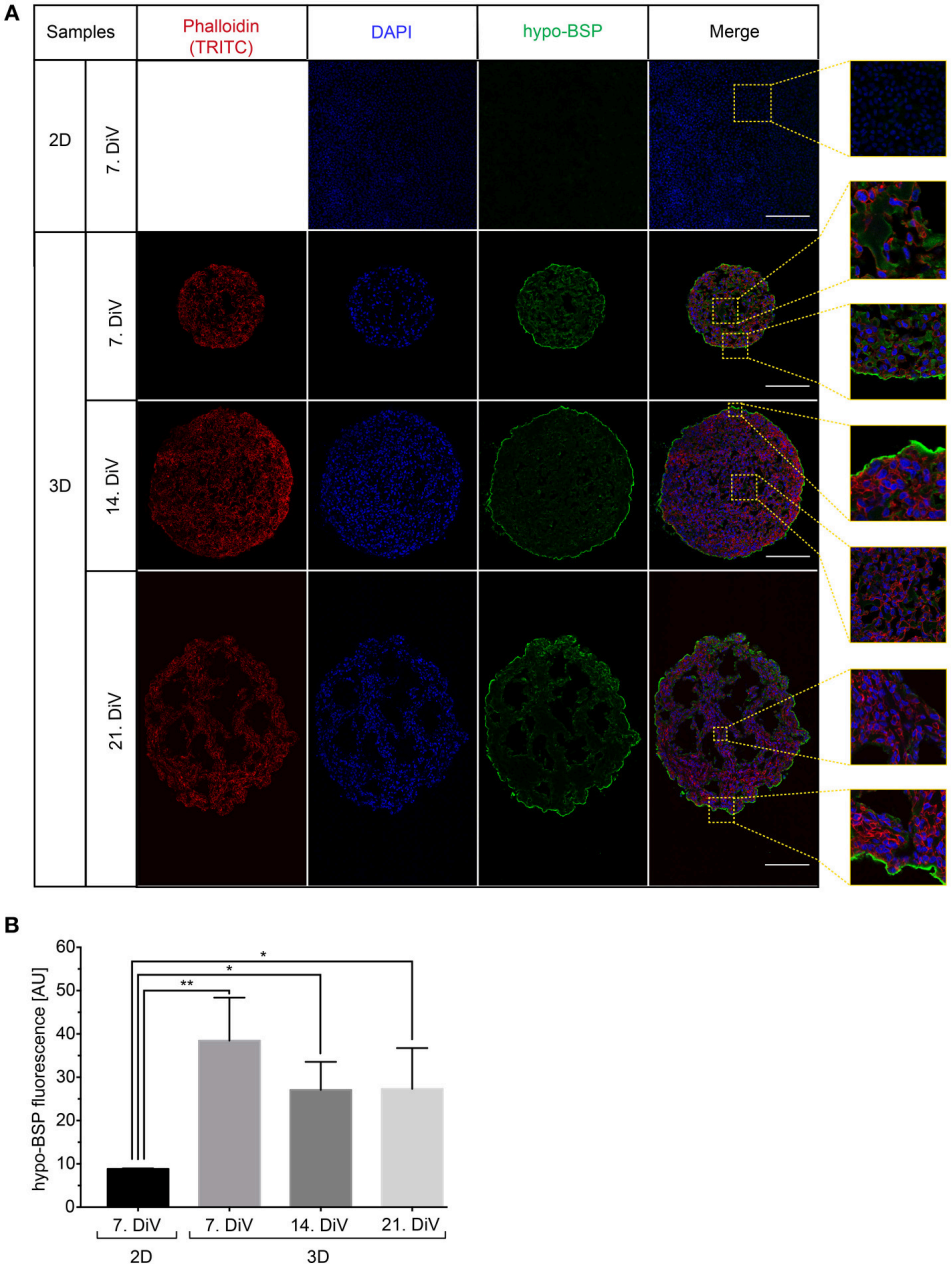
Upregulation of BSP protein levels in young SM3 MDA-MB-231 spheroids is confirmed by immunofluorescence analysis. **(A)** 2D cell cultures were stained with DAPI (blue, cell nuclei) and hypo-BSP antibody (green). Ten micrometer thick cryosections of 7, 14, and 21. DiV spheroids were additionally labeled with Phalloidin-TRITC (red, actin cytoskeleton). Scale bars, 200 μm. Large panels depict optical sections, small panels show details from central and border regions of cultures to illustrate the BSP signal intensity and the enrichment of BSP at the spheroid rims. Detail pictures are taken from the dashed squared areas shown in the large panels. **(B)** Quantitative analysis of confocal images. The graph depicts antibody fluorescence signals as a function of DiV. Statistical analysis was performed with one-way ANOVA followed by Tukey's multiple comparisons test (*n* = 3 for 2D and *n* = 4 for 3D; a total of 148 confocal images were analyzed; ^**^P < 0.01, ^*^P < 0.05, mean ± SD).

To confirm the Western blot findings and to get an insight into the distribution of BSP protein in the spheroids, we next performed immunofluorescence studies with the CD-BSP antibodies. 2D cultures and 10 μm thick cryo-sections of spheroids at different DiV were stained. This resulted in immunofluorescence signals as shown in Figure 4A. While the rat anti-recombinant human CD-BSP antibody did not yield any reliable immunostaining (not shown), the human anti-recombinant human CD-BSP antibody outlined many cell bodies in spheroids with fluorescence intensities being particularly strong at the borders of spheroids (Figure 4A). It is unlikely, that the observed enrichment of BSP fluorescence signals at the spheroid rim was an artifact of the staining procedure, because the strong lining was only found on the actual spheroid borders but not at the tearing edges of spheroid sections ripped during the slicing (Supplementary Figure 4).

To quantify differences in fluorescence intensity on a cellular level, we developed a multistep segmentation routine on the basis of the ImageJ software (https://imagej.nih.gov/ij/) and provide this as a convenient and fully annotated ImageJ macro as a supplement to this study (Data Sheet 1). Results of the new macro are schematically outlined and compared to simple threshold-based segmentation in Supplementary Figure 1. Simple threshold-based segmentation of human anti-CD BSP fluorescence signals was very inefficient and detected primarily the boundaries of spheroids, while the cores of spheroids were hardly segmented (Supplementary Figure 1A). Conversely, the multistep segmentation macro yielded more accurate results (Supplementary Figure 1C). It involved the following steps. First, the raw image was duplicated and all subsequent processing changes were done on these duplicates with no changes on raw data. Next, background noise pixels were removed with a median filter and a high level of intensity was adjusted. The adjusted image was then binarized, dilated, and filtered by watershed. After thresholding and the analyze particles command, detected ROIs were overlaid as a mask onto the raw data, resulting in the final, well-segmented image (Supplementary Figure 1C, lower right). In comparison to the right panel of Supplementary Figure 1A, which shows the same image analyzed with simple thresholding, a clear difference in the quality of segmentation is evident: While the novel routine was good to detect most internal cellular borders, the simple method found almost exclusively the strongly stained spheroid border and a couple of noise pixels (indicated by arrowheads in Supplementary Figures 1A,C). As shown in Supplementary Figure 1B, the same algorithm was also useful for segmenting DAPI-stained cell nuclei in the same samples.

By using the new macro, we then quantitatively compared anti-CD BSP immunofluorescence signals from samples taken at different DiV (Figure 4B). This showed similar trends in hypo-BSP expression as those obtained by Western blotting. Indeed, hypo-BSP immunofluorescence signals were 4.3-fold higher in 7. DiV spheroids than in 2D cultures (Figure 4B) and declined in 14. and 21. DiV spheroids as compared to 7. DiV spheroids (Figure 4B).

### Cell Proliferation and Apoptosis Profiles Do Not Correlate to BSP Expression

**Figure 5:**
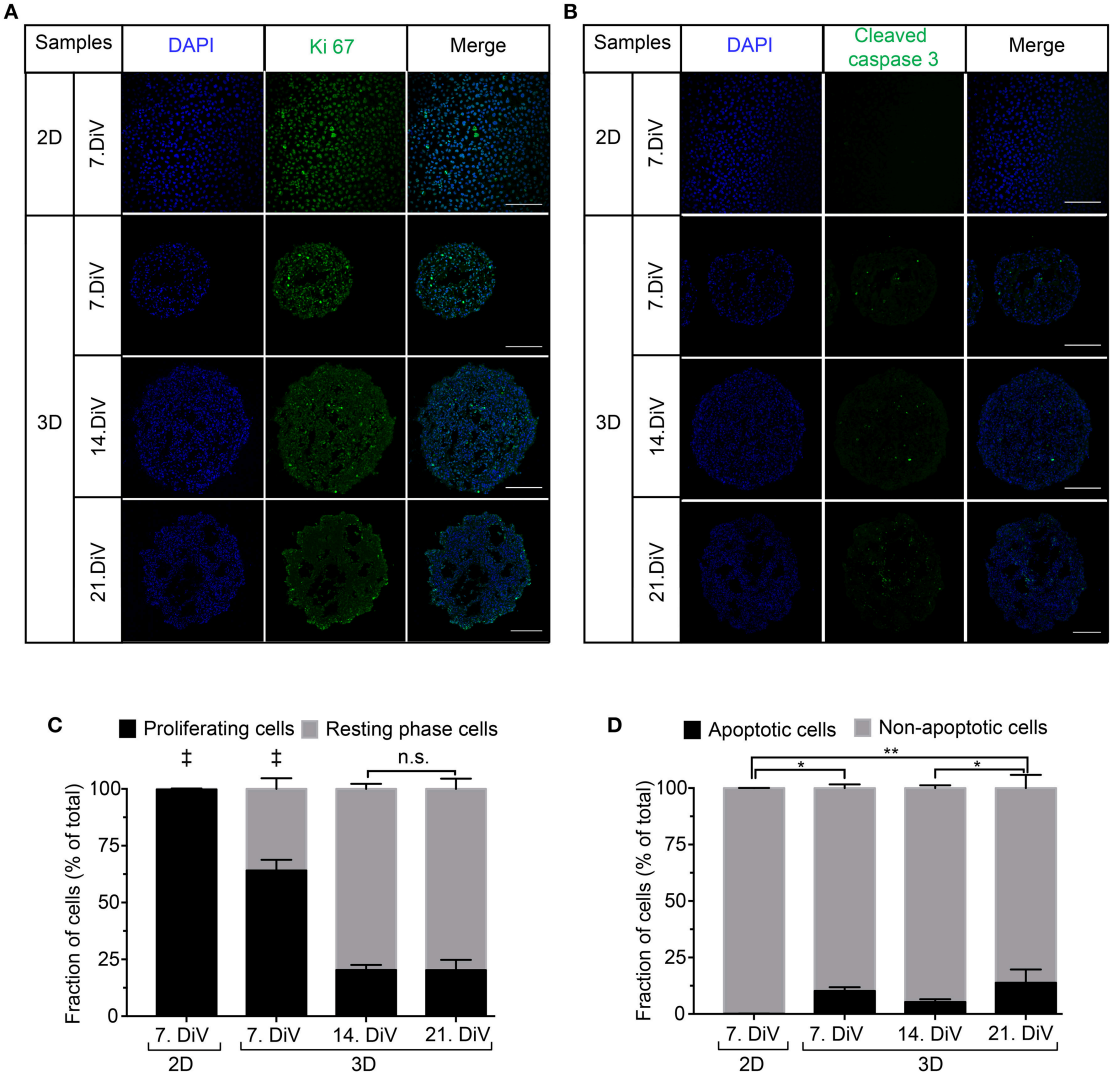
Cell proliferation decreases in MDA-MB-231 spheroids with increasing DiV. **(A,B)** Monolayer cell culture and 10 μm thick cryosections of 7., 14., and 21. DiV spheroids were stained with DAPI (blue) and immunolabeled for either Ki 67 (**A**, proliferation marker, green) or Cleaved Caspase 3 (**B**, apoptosis marker, green). Images show single confocal slices of representative samples. Scale bars, 200 μm. **(C,D)** Quantitative analysis of confocal images. Statistical analysis was performed with one-way ANOVA followed by Tukey's multiple comparisons test. **(C)** Graphs depict mean ± SD (*n* = 3 for 2D and 3D; a total of 85 confocal images were analyzed; ‡ = Significant difference to all other values). **(D)** Graphs depict mean ± SD (*n* = 3 for 2D and 3D; a total of 81 confocal images were analyzed; ^**^P < 0.01, ^*^P < 0.05).

To investigate a potential correlation between BSP expression and physiological processes, we first addressed the occurrence of cell proliferation and apoptosis in 2D and spheroid samples. Therefore, 2D-cultures and spheroid sections were immunostained with anti-Ki 67 (Figures 5A,C) and anti-Cleaved Caspase 3 antibodies (Figures 5B,D). Subsequently, the numbers of immune-positive cells were determined using the newly developed ImageJ segmentation macro. This showed more proliferating cells in 2D cultures of MDA-MB-231 cells than in 7. DiV spheroids (Figure 5C). Moreover, the fraction of proliferating cells decreased significantly from 7. DiV to 14. DiV spheroids and then remained stable (Figure 5C). Conversely, the number of apoptotic cells was extremely low in 2D cell culture, increased significantly in 7. DiV spheroids and from there remained similar with slight but significant changes between 14. and 21. DiV (Figure 5D). In conclusion, neither the profile of numbers of proliferating nor of apoptotic cells fit to the observations for BSP expression.

### Expression of TGFβ1 Correlates With That of BSP in Different Conditions

**Figure 6:**
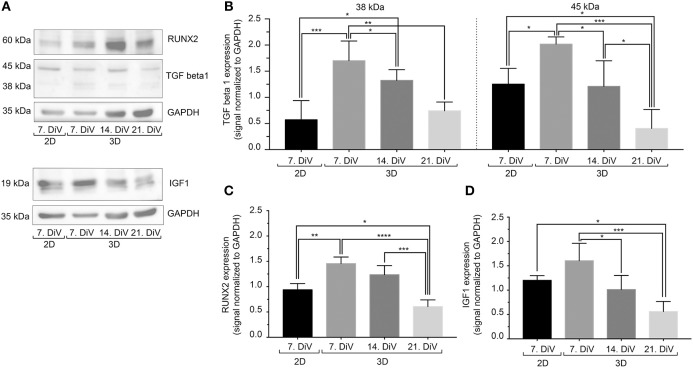
In untreated SM3 spheroids, expression profiles of TGFβ1, IGF-1, and RUNX2 are similar to that of BSP. Equal amounts of protein of lysates from monolayer cells (2D) as well as 7., 14., and 21. DiV spheroids (3D) were loaded on SDS-PAGE and Western blots were performed. **(A)** Representative Western blot profiles for TGFβ1, RUNX2, IGF-1, and GAPDH (loading control). **(B-D)** Quantitative analyses of Western blot bands. Statistical analysis was performed with one-way ANOVA followed by Tukey's multiple comparisons test (*n* = 4, ^****^P ≤ 0.0001, ^***^P < 0.001, ^**^P < 0.01, ^*^P < 0.05, mean ± SD).

In breast cancer cells, including MDA-MB-231, BSP expression is mediated by the transcription factor, RUNX2 (70). Further, regulation of RUNX2 abundance and transcriptional activity appear to be under control of TGFβ1 (71) and IGF-1 (55). To address the regulation of BSP expression from that point of view, three different types of approaches were performed. First, cell lysates were prepared from 7. DiV 2D cultures as well as from 7., 14., and 21. DiV spheroids and were then subjected to Western blotting. As depicted in Figures 6A,B, TGFβ1 signals in the range between 38–50 kDa, likely reflecting different intracellular maturation stages of pre-pro- and pro-TGFβ1 (72), were rather low in the 2D culture samples, peaked in the 7. DiV spheroids and then declined in older spheroids. Levels of RUNX2 were similar in 2D and young spheroids and then decreased with augmenting spheroid age (Figures 6A,C). Finally, also pre-pro- (25 kDa) and pro-IGF-1 (17 kDa) (73) followed a similar expression profile (Figures 6A,D). Thus, under this condition the expression profiles of TGFβ1, IGF-1, and RUNX2 correlated well with that of BSP, consistent with the reported signaling axis. Second, 6. DiV spheroids were treated for 48 h with 0 or 1 μM of Paclitaxel. Upon immunofluorescence staining of these samples, the following observations were made. First, Paclitaxel treatment induced apoptosis, but did not alter the fraction of Ki-67 positive cells (Supplementary Figure 5). Next, the cytostatic reduced the immunofluorescence signals of both, BSP and TGFβ1, but not that of IGF-1 (Supplementary Figure 5). As a third approach, the expression profiles were also examined during prolonged culture times of MDA-MB-231 co-culture spheroids with CCD-1137Sk fibroblasts. Similar to the SM3 spheroids, also the co-culture spheroids showed the highest BSP fluorescence signals in the peripheral cells (Supplementary Figure 6A). On average, the fluorescence signals were 32.8% ± 9.0% brighter there than in the spheroid centers. As observed in the MDA-MB-231 monocultures, both, BSP and TGFβ1 immunofluorescence signals decreased with increasing DiV (Supplementary Figure 6). Conversely, IGF-1 signals increased with higher DiV (Supplementary Figure 6). In summary, these data show a consistent correlation between the expression of BSP and TGFβ1, while IGF-1 profiles showed a more inconsistent behavior.

## Discussion

Accumulating evidence describes BSP as an important protein for tumorigenesis and metastasis in breast cancer (45). However, expression and function of BSP appears to be highly dependent on the appropriate cellular environment. In particular, while abundance of BSP is high in metastatic breast and bone tissues, it is low or absent in 2D cultures of cells derived from these entities. Therefore, in order to address regulation and function of BSP protein *in vitro*, appropriate 3D cell culture systems were needed. Here, we first set up and characterized two long-term spheroid cell culture models of MDA-MB-231 breast cancer cells. Then, these models were used to study BSP expression in 2D vs. 3D longitudinal profiles and upon cytostatic treatment. These approaches revealed a transient upregulation of BSP upon 3D culturing as well as a good correlation of TGFβ1 expression with that of BSP.

It is well-known that cancer cell lines show different characteristics depending on 3D culture conditions (63, 74, 75). Previous studies reported that the ratio between hydrogel and Matrigel (76, 77) or the stiffness of the mixture (78) could influence 3D cell growth and morphology. MDA-MB-231 cells are no exception and they were found to form different types of 3D aggregates, e.g., round (63) or stellate (79). Here, we concentrated on culture protocols for reaching regularly round, linearly growing, and long-term stable spheroids. Of the ones tested, a sandwich-type culture protocol was optimal to mediate consistent growth of large-sized spheroids for more than 1 month (depicted here is up to 24 DiV). These results and the similar findings we have made in this study for the prostate cancer cell line, PC-3, corroborate our previous work, where, depending on 3D cell culture technique and initial seeding density, differently sized spheroids of SCC-4 tongue cancer cells were obtained (80). With respect to spheroid formation and linear long-term growth kinetics of MDA-MB-231 cells, protein-rich BME (14–16 mg/ml) was superior to Matrigel (9–12 mg/ml) in the present study. Both matrices are derived from Engelbreth-Holm-Swarm mouse sarcoma, and contain growth factors (EGF, FGF, TGFβ, IGF) (81), ECM proteins (laminin, collagen IV, entactin) (82), proteases (MMP-2, MMP-9) (83), and perlecan (84). Yet, BME and Matrigel yielded differently sized spheroids of LNCaP prostate cancer cells (85). Furthermore, cancer cells injected with high-protein content Matrigel displayed significantly accelerated tumorigenesis *in vivo* when compared to low concentrated Matrigel (86). In accordance with previous studies, our SM3 spheroid culture model showed similar growth characteristics as tumor nodules *in vivo*, which were generated by injection of MDA-MB-231 breast cancer cell suspension mixed with 100 μl Matrigel (87). Notably, during the entire observation period, SM3 spheroid growth was almost linear although the number proliferative cells decreased after 7. DiV. To explain this apparent discrepancy, two aspects might be relevant. First, although cell proliferation significantly dropped after 7. DiV it still slightly outweighed the fraction of apoptotic cells. Thus, considering these as the principal sources for cell number, there should be a net increase in spheroid size even at 14. and 21. DiV. Second, the growth curves in Figure 1C simply reported the spheroid area from the outside. As can be seen in Figures 3, 4, though, the inside of the older SM3 spheroids increasingly displayed empty regions, which were likely due to cell death or outward movement of cells away from the core zone. In combination, we think, these two points could well account for the observed linear growth of SM3 culture spheroid areas.

The relevance of an appropriate ECM for the 3D-growth of MDA-MB-231 cells was further supported by our co-culture data, where MDA-MB-231 cells were grown in 3D together with ECM producing CCD-1137Sk foreskin fibroblast cells. Although spheroid condensation took longer in these co-cultures as compared to the SM3 model and although many migrating cells were observed, spheroids from the co-culture model exhibited steady long-term growth over the test period of 25 DiV. Potentially, such a model could be used to study tumor-stroma interactions, but it would also bear the drawback that biochemical analyses will be based on a mixed cellular composition. Interestingly, in both, SM3 as well as co-culture spheroids, BSP was enriched in or on the external cells. Since these cells were well-aligned in the SM3 cultures and more unordered in co-cultures, peripheral BSP enrichment was more easily seen in the SM3 cultures, but it was present in both types of spheroids. Considering the proposed role of BSP in metastatic cells, i.e., modulating the ECM for niche formation as a function of TGFβ1 (88), this peripheral accumulation might serve as diagnostic feature, which would not be available in 2D cultures. Taken together, our results confirm that the cells cultured in 3D with Matrigel or BME may serve as a reliable *in vitro* model for the study of long-term 3D growth of breast and prostate cancer cells, with BME SM3 spheroids being larger on average.

Using this model, we demonstrated that BSP expression was upregulated in young MDA-MB-231 spheroids as compared to 2D cultures. In older spheroids, though, BSP expression returned to lower values. These results were first obtained with Western blot analysis using two different anti-BSP antibodies, which primarily detected either the hypo-glycosylated or the mature form of BSP. For the hypo-BSP recognizing antibody, these findings could be confirmed by immunofluorescence analysis. Previously, conditional knockdown of BSP in 2D cell cultures was reported to cause increased apoptosis and reduced cell proliferation as well as decreased bone metastasis in a mouse xenograft model (89). Thus, we first addressed a putative correlation between BSP expression and apoptosis or proliferation. With respect to apoptosis, almost no Cleaved Caspase-3 positive cells were found in 2D while at all tested spheroid ages, the fraction of apoptotic cells varied between only six and fourteen percent. Thus, there was no similarity to the BSP expression profile arguing against a simple negative correlation between BSP expression and apoptotic cell death. As for cell proliferation, a gradual decline of Ki-67 positive cells was found upon prolonged culture in 3D, reminiscent of the BSP expression profile. However, proliferation was highest in the 2D cultures, when BSP was very low. Therefore, also between BSP expression and cell proliferation a good fit was not evident.

To address further options, we next concentrated on the link between BSP and metastasis. Indeed, knockdown of BSP was shown to reduce metastatic potential of cells to bone (89) and lung in mice (88). Furthermore, impairment of BSP expression decreased basal and TGFβ1-stimulated activation of MMPs and reduced degradation of type I or type IV collagen (88). Amongst other processes, metastasis competence involves epithelial-mesenchymal transition (EMT) enabling migration and extravasation potential of cancer cells, as well as a homing process, where an adequate metastatic niche provides appropriate ECM-based interactions between cancer cells and target tissue. In the context of metastatic breast cancer, IGF-1 and TGFβ1 are both known to be important regulators of EMT (90, 91). However, while IGF-1 abundance is reported to correlate with both, breast cancer cell proliferation and metastatic potential (91), TGFβ1 is thought to promote primarily ECM and niche formation, cytoskeletal reorganization, cell motility, and invasion (90, 91), but not cell proliferation. Indeed, according to the “TGFβ1 paradox” concept, this cytokine acts as a potent growth inhibitor in healthy epithelia and during early tumor phases while at later stages it promotes the metastatic process by supporting invasion of cancer cells and metastatic niche formation (90). The data of the present study are consistent with an axis involving TGFβ1, IGF-1, and RUNX2 in the joint regulation of BSP levels. However, while TGFβ1 and BSP showed rather similar expression profiles in SM3 and co-culture maturation as well as upon Paclitaxel treatment of SM spheroids, the IGF-1 expression profile was similar to that of BSP only in untreated SM3 cultures. This suggests, that differential pathways might be involved in the regulation of BSP expression.

## Conclusion

In summary, we optimized methods for long-term culture of triple-negative MDA-MB-231 breast cancer cells in 3D and showed the influence of fibroblasts and different extracellular matrix compounds, such as Matrigel and BME, on spheroid formation. A novel human anti-BSP monoclonal antibody was characterized and found to display specificity to hypo-BSP. Using these 3D mono- and co-cultures in different experimental paradigms, a consistent correlation between the expression of BSP and TGFβ1 could be confirmed. In contrast, there was only a partial connection of BSP abundance to apoptosis, proliferation, and IGF-1.

## Author Contributions

VR, FK, and JK executed, analyzed, interpreted experiments. VR wrote paper. MH and RR planned experiments, procured funding, and wrote paper.

### Conflict of Interest Statement

Immundiagnostik AG provided anti-BSP antibodies used in the present work. This company is active partner in the public private partnership M2OGA (03FH8I02IA) within the framework “Starke Fachhochschulen—Impuls für die Region” (FH-Impuls) that is funded by the German Federal Ministry of Research. At no point in this study, Immundiagnostik AG interferred or influenced the work; they did not plan the experiments shown and were not involved at any point in data interpretation or writing of the manuscript.
