# Supplementary material for: Bone Sialoprotein Shows Enhanced Expression in Early, High-Proliferation Stages of Three-Dimensional Spheroid Cell Cultures of Breast Cancer Cell Line MDA-MB-231
Source: Front Oncol. 2019 Feb 5;9:36. doi: 10.3389/fonc.2019.00036 (PMC6370714; doi:10.3389/fonc.2019.00036)
Supplement: Supplementary file 1 [file Table_1.DOCX]

**Supplementary Table 1**. List of antibodies and dyes used for western blot (WB) and immunofluorescence studies (IF).

| **Targeted antigen/ cells** | | **Manufacturer/ Catalog No.** | **Host/ Isotype/ Clone** | **Working Dilution** | |
| --- | --- | --- | --- | --- | --- |
|  |  |  |  | **WB** | **IF** |
| **Primary antibodies:** | | | | | |
| Apoptotic cells | Cleaved Caspase 3 | Cell signaling / 9661 | Rabbit/  pAb | –– | 1:500 |
| Carbohydrate deficient bone sialoprotein | Recombinant CD-BSP (aa 108-122) | Immunodiagnostik AG, Germany | Human/  IgG/ mAb | 1:250 | 1:250 |
|  | Recombinant CD-BSP (aa 108-122) TGC-9 | Immunodiagnostik AG, Germany / 4217.VP | Rat/ IgG1/mAb | 1:250 | –– |
| Collagen1 | Anti-Collagen Type 1 | Rockland Immunochemicals Inc. / 600-401-103-0.5 | Rabbit/IgG/pAb | –– | 1:150 |
| GAPDH | Recombinant GAPDH | Thermo Fisher Scientific / MA5-15738 | Mouse/ IgG1/ mAb | 1:10000 | –– |
| IGF1 | Anti IGF1 | Thermo Fisher Scientific / PA5-27207 | Rabbit/IgG/pAb | 1:1000 | 1:100 |
| Proliferating cells | Ki67 | Merck / AB9260 | Rabbit/  pAb | –– | 1:500 |
| RUNX2 | Anti RUNX2 | Cell signaling / D1L7F | Rabbit/  mAb | 1:200 | –– |
| TGF-ß1 | Anti TGF-ß1 | Abcam/ab92486 | Rabbit/IgG/pAb | 1:1000 | 1:500 |
| **Secondary antibodies:** | | | | | |
| Goat anti human (H+L), Alexa Fluor 488 | | Thermo Fisher Scientific / A11013 | Goat/ IgG/ pAb | –– | 1:1000 |
| Goat anti human HRP | | Dianova /  109-035-098 | Goat/  IgG/ pAb | 1:50000 | –– |
| Goat anti mouse HRP | | Sigma-Aldrich / A2304 | Goat/  IgG/ pAb | 1:10000 | –– |
| Goat anti rabbit (H+L),  Alexa Fluor 488 | | Invitrogen / A21206 | Goat/ IgG/ pAb | –– | 1:1000 |
| Goat anti rabbit (H+L),  Alexa Fluor 647 | | Invitrogen / A21246 | Goat / IgG/ pAb | –– | 1:1000 |
| Rabbit anti rat HRP | | Sigma-Aldrich / A5795 | Rabbit / IgG/ pAb | 1:10000 | –– |
| **Dyes:** | | | | | |
| Actin, Cytoskeleton | Rhodamine  Phalloidin (TRITC) | Thermo Fisher Scientific | –– | –– | 1:1000 |
| Nucleus | DAPI | Thermo Fisher Scientific | –– | –– | 1:1000 |
