# Supplementary figures and images for: Bone Sialoprotein Shows Enhanced Expression in Early, High-Proliferation Stages of Three-Dimensional Spheroid Cell Cultures of Breast Cancer Cell Line MDA-MB-231
Source: Front Oncol. 2019 Feb 5;9:36. doi: 10.3389/fonc.2019.00036 (PMC6370714; doi:10.3389/fonc.2019.00036)

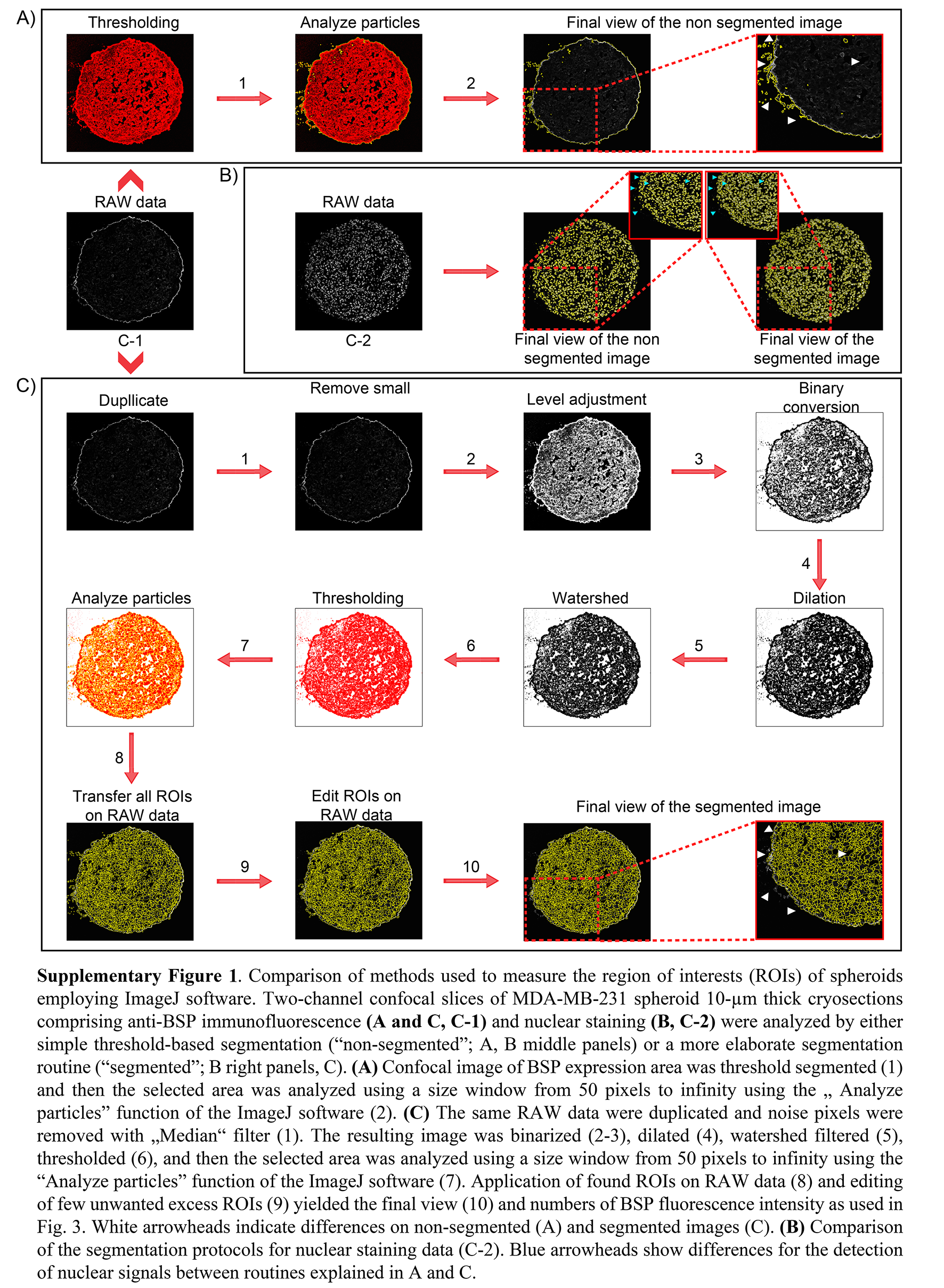

Supplement: Supplementary file 3 [file Image_1.TIF]

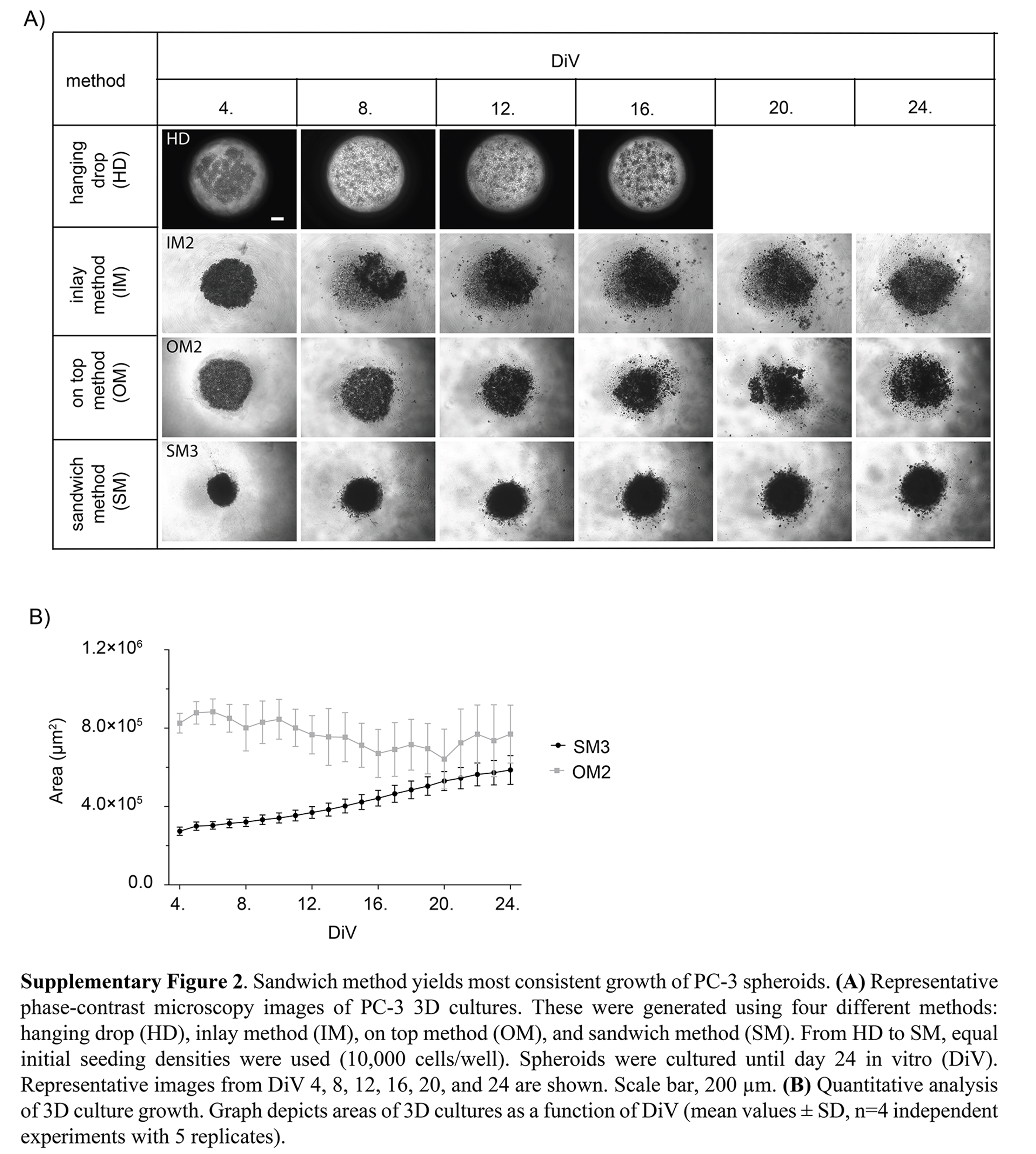

Supplement: Supplementary file 4 [file Image_2.TIF]

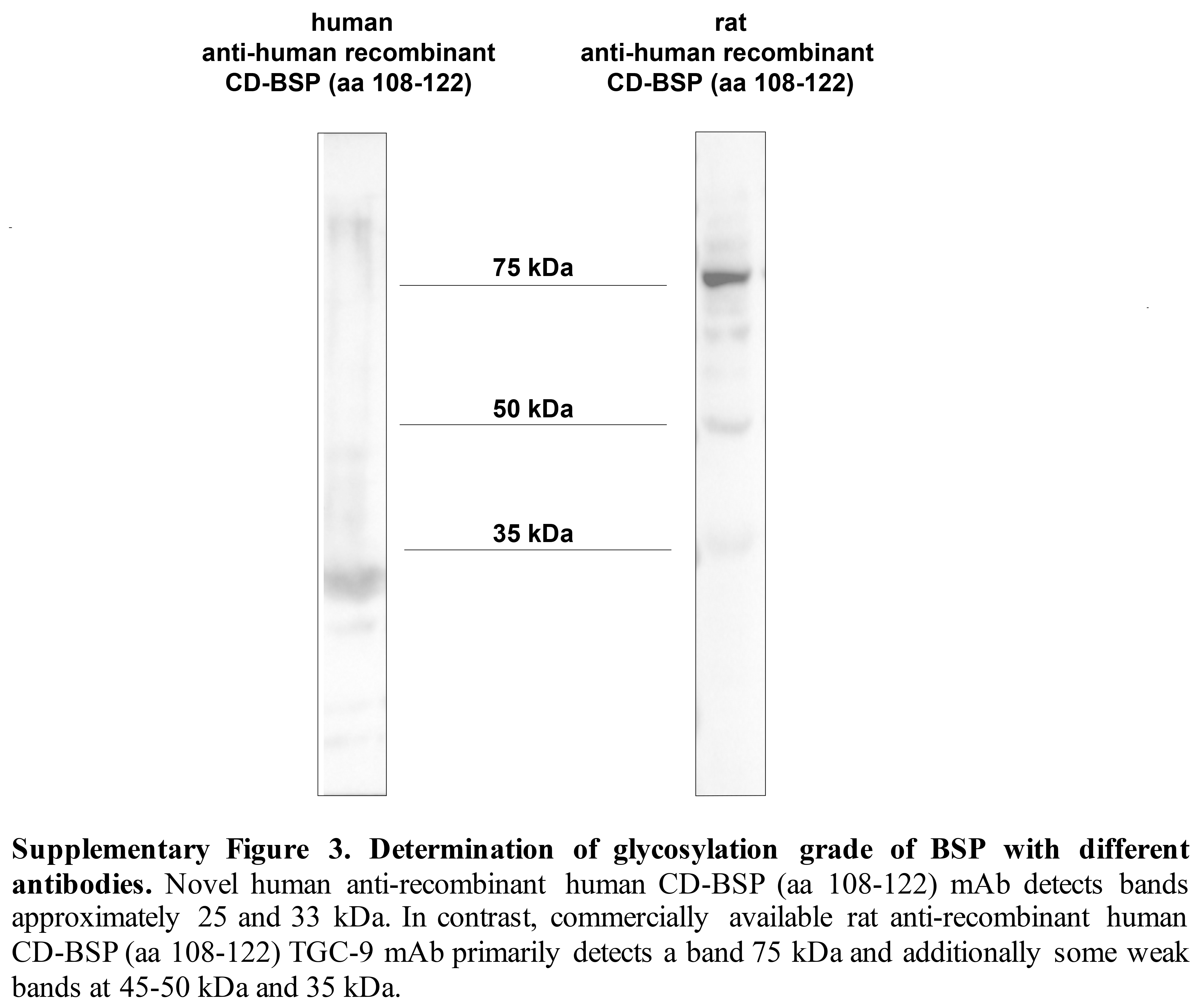

Supplement: Supplementary file 5 [file Image_3.TIF]

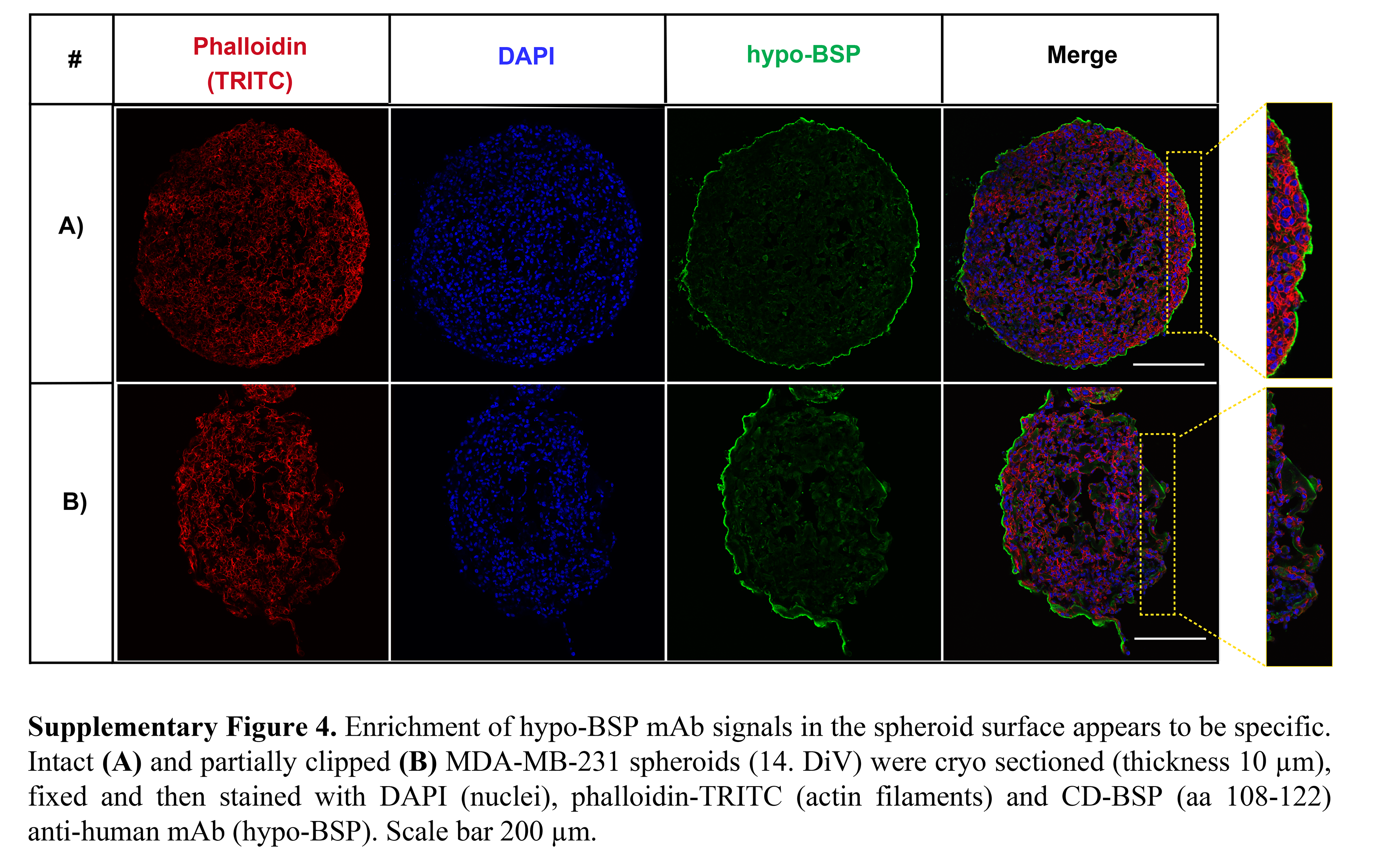

Supplement: Supplementary file 6 [file Image_4.TIF]

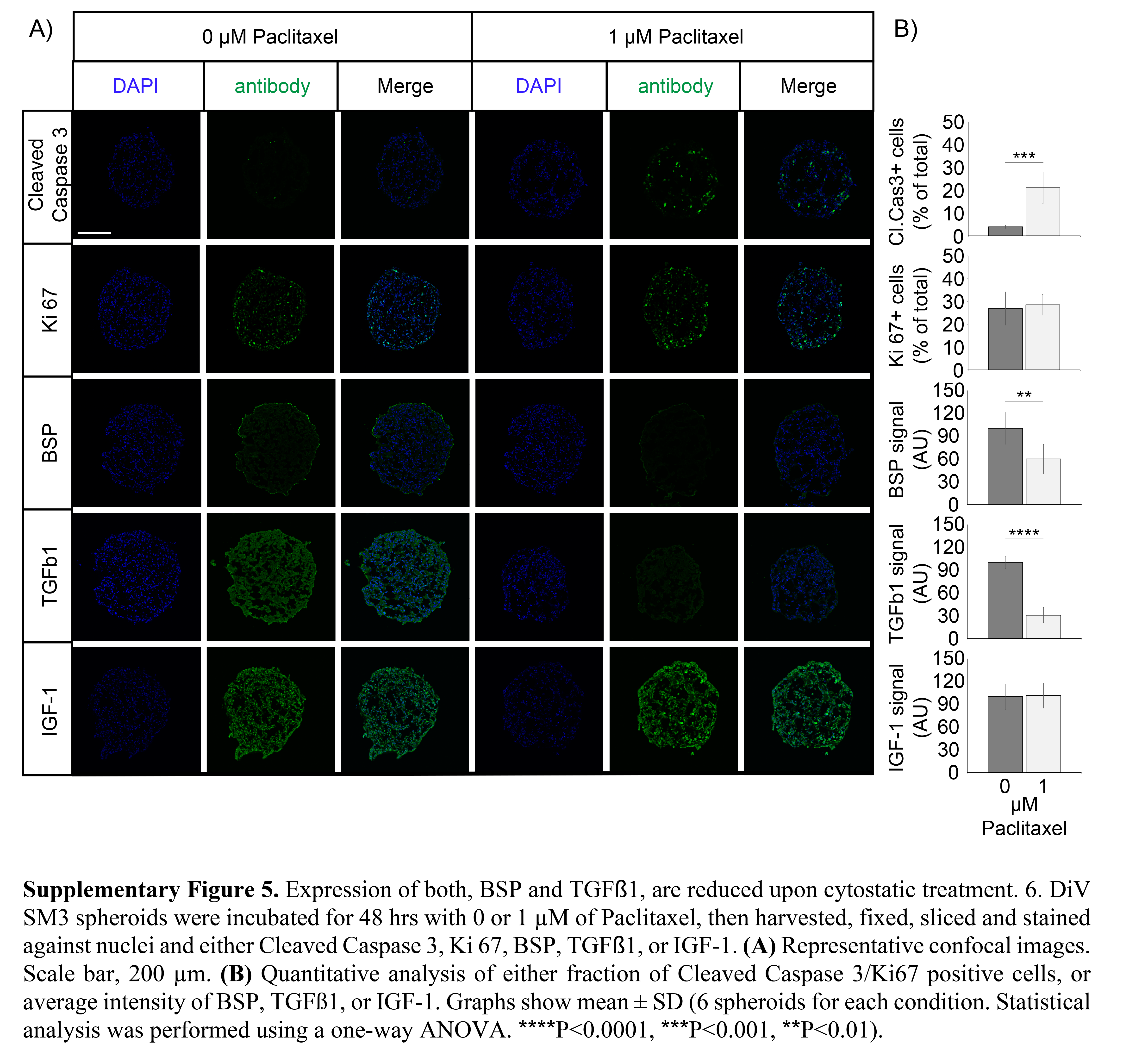

Supplement: Supplementary file 7 [file Image_5.TIF]

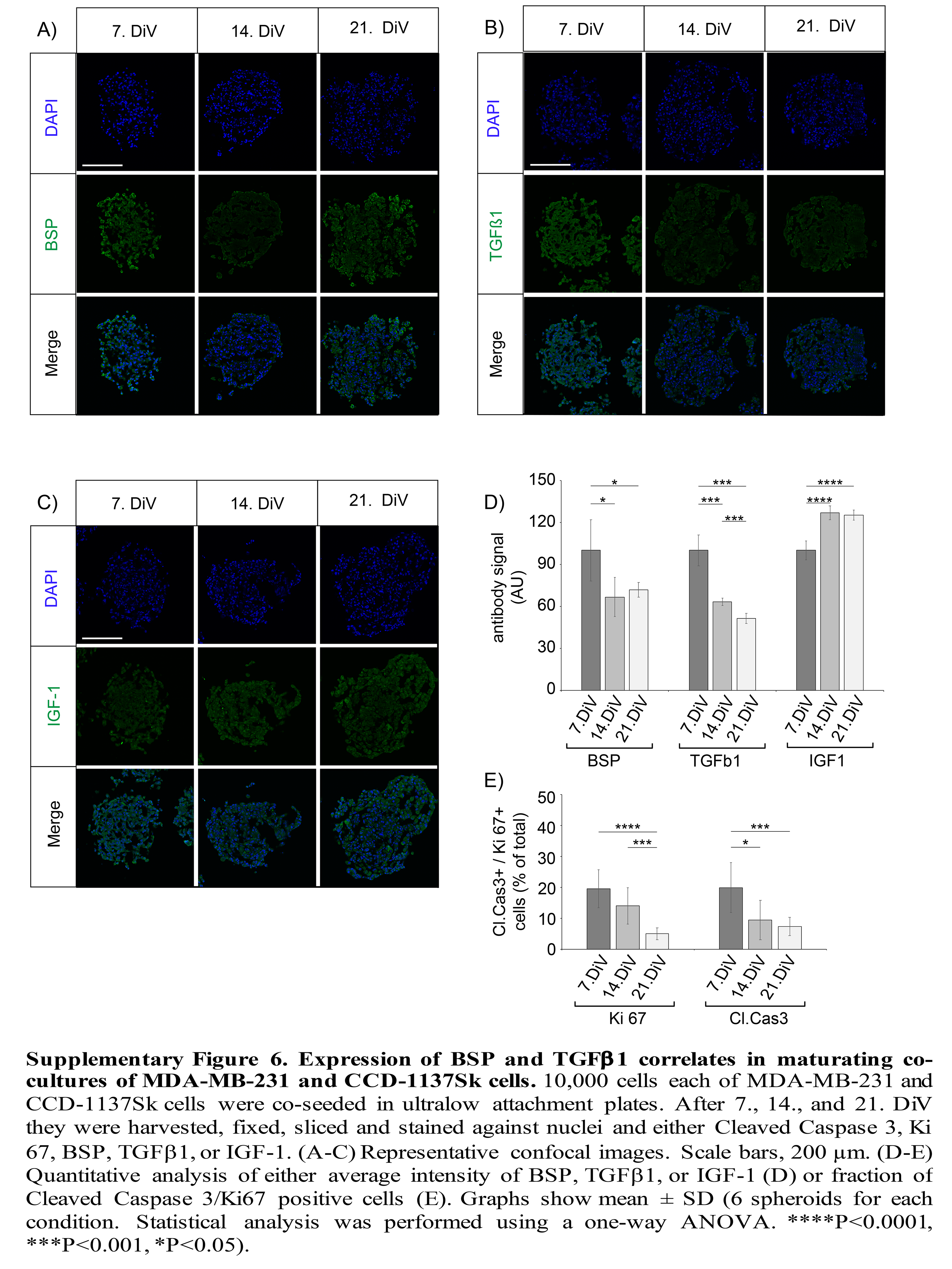

Supplement: Supplementary file 8 [file Image_6.TIF]
